# Pulmonary angiomatoid fibrous histiocytoma invading the pulmonary vein with intracranial metastasis: a case report

**DOI:** 10.3389/fonc.2026.1907177

**Published:** 2026-08-10

**Authors:** Yifei Tu, Yurun Xia, Zhen Wang

**Affiliations:** 1 Zhejiang Chinese Medical University, Hangzhou, Zhejiang, China; 2 Shangyu People’s Hospital of Shaoxing, Shaoxing, Zhejiang, China; 3 Department of Radiology, Affiliated Hangzhou First People’s Hospital, School of Medicine, Westlake University, Hangzhou, Zhejiang, China; 4 Zhejiang Key Laboratory of Zero Magnetic Medicine, Affiliated Hangzhou First People’s Hospital, School of Medicine, Westlake University, Hangzhou, Zhejiang, China

**Keywords:** angiomatoid fibrous histiocytoma, case report, intracranial metastasis, pulmonary vein invasion, tumor

## Abstract

Pulmonary mesenchymal tumors are extremely rare. Angiomatoid fibrous histiocytoma (AFH) originating in the lungs is particularly uncommon, with clinical manifestations similar to those of lung cancer, which often leads to misdiagnosis. We report the case of a 77-year-old male patient who presented with a left hilar mass following trauma. Imaging revealed a mass resembling lung cancer with bronchial and vascular invasion. Bronchoscopic biopsy and immunohistochemistry confirmed an intermediate-type pulmonary AFH. Because of multiple comorbidities, the patient was not eligible for surgical treatment. Six months later, he developed multiple brain metastases with hemorrhage and edema, indicating a high malignant potential and distant metastatic capability. This case enriches the data on pulmonary AFH in older patients, emphasizing its importance in the differential diagnosis of hilar masses and the associated diagnostic and treatment challenges. Moreover, it highlights the need for early identification and standardized follow-up of incidental pulmonary lesions supported by multidisciplinary collaboration for accurate diagnosis. Given the morphological similarity of pulmonary AFH to various spindle cell tumors, immunohistochemistry and molecular pathology are key to its diagnosis. To the best of our knowledge, this is one of very few reported cases of brain metastasis arising from primary pulmonary AFH, emphasizing its potential aggressiveness. Data accumulation, improved diagnostic and treatment standards, and comprehensive management strategies for older complex patients is required to enhance treatment outcomes and quality of life. This report provides a valuable reference for the clinical spectrum and metastatic mechanism of pulmonary AFH.

## 1 Introduction

Angiomatoid fibrous histiocytoma (AFH), initially described by Enzinger in 1979, was classified by the World Health Organization in 2020 as a “differentiation uncertain tumor” with moderate biological potential and unclear differentiation. AFH accounting for 0.3% of soft tissue tumors. While the prognosis for most AFH patients is favorable, approximately 15% experience local recurrence, and 1-5% develop distant metastases (1). Primary pulmonary cases are scarcely reported in the literature (2). AFH typically occurs in adolescents and young adults, whereas pulmonary involvement is more common in middle-aged and older patients. Its pathogenesis and biological behavior remain unclear, and its clinical and imaging features often overlap significantly with those of common lung cancers and other mesenchymal tumors (2). Herein, we report the case of a 77-year-old male patient with a rare primary lesion in the left lower lobe adjacent to the mediastinum. The imaging findings resembled lung cancer, which could lead to misdiagnosis. This case highlights the need for vigilance in older populations and further enriches the epidemiological data on pulmonary AFH.

Rare pulmonary spindle cell tumors lack specificity in clinical and imaging presentations. They manifest as hilar enlargement, mediastinal masses, and bronchial or vascular invasion, and are easily confused with common malignant tumors such as non-small cell lung cancer (3). Therefore, the early identification and accurate diagnosis of pulmonary AFH are highly challenging. Because of their histological similarity to various spindle cell tumors, immunohistochemistry and molecular pathology are crucial for definitive and differential diagnoses (4, 5). We present the bronchoscopic biopsy and immunohistochemistry findings that confirmed the intermediate-type angiomatoid fibrous histiocytomatous nature of the tumor, emphasizing the importance of multidisciplinary collaboration in diagnosing rare pulmonary tumors.

Although pulmonary AFH is often low-grade malignant, this case presented with multiple brain metastases accompanied by hemorrhage and significant edema, indicating high malignant potential and distant metastatic capability (6–8). Reports of pulmonary AFH with brain metastasis are extremely rare in the literature; therefore, this case is a valuable reference for the metastatic behavior and clinical evolution of primary pulmonary AFH. Moreover, it alerts clinicians to the potential invasiveness and metastatic risk of unidentified spindle cell tumors in the lung.

The objective of this report is to describe the clinical presentation, diagnostic challenges, and clinical evolution of distant brain metastasis in a patient with pulmonary AFH.

## 2 Patient information

A 77-year-old male patient was admitted to Shangyu People’s Hospital in Shaoxing three years ago because of left waist pain caused by a fall. A chest computed tomography (CT) scan suggested left hilar enlargement, and a contrast-enhanced CT scan was recommended; however, the patient did not undergo further examination. Two years ago, he was hospitalized because of the discovery of atrial fibrillation, a complete right bundle branch block, and ST-T segment changes during a health checkup.

The patient had a history of hyperlipidemia but did not take medication regularly; hyperglycemia, with long-term use of Compound Danshen tablets; herpes zoster; residual neuropathic pain; long-term use of gabapentin; sleep disturbance; intermittent use of estazolam; and history of lower limb varicose vein surgery. He had no history of oncogenesis or family genetic diseases.

### 2.1 Clinical findings

The health checkup on the day of admission showed that the patient was conscious, with a heart rate of 95 beats/min, an absolutely irregular cardiac rhythm, no murmurs, coarse respiratory sounds in both lungs, and no obvious symptoms such as chest pain, hemoptysis, fever, headache, syncope, or limb movement disorders. Pigmentation and varicose veins were observed in both lower limbs. The vital signs recorded upon admission are presented in **Table 1**.

**Table 1:** Physical examination data on admission.

Tests	Result	Reference range	Interpretation
Vital signs			
Blood pressure (mm Hg)	139/84	< 120/80	**High**
Heart rate (beats/min)	84	60–100	Normal
Respiratory rate (breaths/min)	19	12–20	Normal
Temperature (°C)	36.7	37	Normal

Bold values denote abnormal biochemical laboratory test results.

### 2.2 Diagnostic assessment

The detailed results of the hematological, biochemical, coagulation, and tumor marker tests are summarized in **Table 2**.

**Table 2:** Laboratory test results on admission.

Tests	Result	Reference range	Interpretation
Hematology			
White blood cell count (×10^9^/L)	5.6	3.5–9.5	Normal
Red blood cell count (×10¹²/L)	4.17	4.3–5.8	**Low**
Hemoglobin (g/L)	130	130–175	Normal
Platelet count (×10^9^/L)	129	125–350	Normal
C-reactive protein (mg/L)	14.7	0–8	**High**
Blood biochemistry			
Urea nitrogen (mmol/L)	6.93	2.3–7.8	Normal
Serum creatinine (μmol/L)	73.7	44–106	Normal
Uric acid (μmol/L)	404	149–416	Normal
Albumin (g/L)	35.4	40–55	**Low**
Alkaline phosphatase (U/L)	70.3	45–125	Normal
Alanine aminotransferase (U/L)	32.2	9–50	Normal
Aspartate aminotransferase (U/L)	27.9	15–40	Normal
Potassium (mmol/L)	3.76	3.5–5.3	Normal
Sodium (mmol/L)	144	137–147	Normal
Chloride (mmol/L)	109	99–110	Normal
Calcium (mmol/L)	2.04	2.13–2.63	**Low**
Magnesium (mmol/L)+	0.92	0.7–1.1	Normal
Glucose (mmol/L)	6.61	3.89–6.11	**High**
Total cholesterol (mmol/L)	3.55	3.29–4.99	Normal
Triglyceride (mmol/L)	1.71	0.11–2.03	Normal
Coagulation profile			
Prothrombin time (s)	19.7	10.5–14	**High**
Activated partial thromboplastin time (s)	42.9	20–50	Normal
Fibrinogen (g/L)	2.9	2–4	Normal
Thrombin time (s)	16.2	12–21	Normal
Tumor markers			
Carcinoembryonic antigen (ng/mL)	5.78	0–5	**High**
Alpha-fetoprotein (ng/mL)	1.42	0–13.6	Normal
Carbohydrate antigen 125 (U/mL)	27.76	<35	Normal
Carbohydrate antigen 199 (U/mL)	15.71	0–39	Normal
Carbohydrate antigen 724 (U/mL)	0.76	0–6.9	Normal
Cytokeratin 19 fragment (ng/mL)	1.71	0–3.3	Normal
Neuron-specific enolase (ng/mL)	13.43	0–16.3	Normal
Total prostate-specific antigen (ng/mL)	1.08	<4	Normal

Bold values denote abnormal biochemical laboratory test results.

Urinalysis and stool occult blood test results were negative.

**Figure 1:**
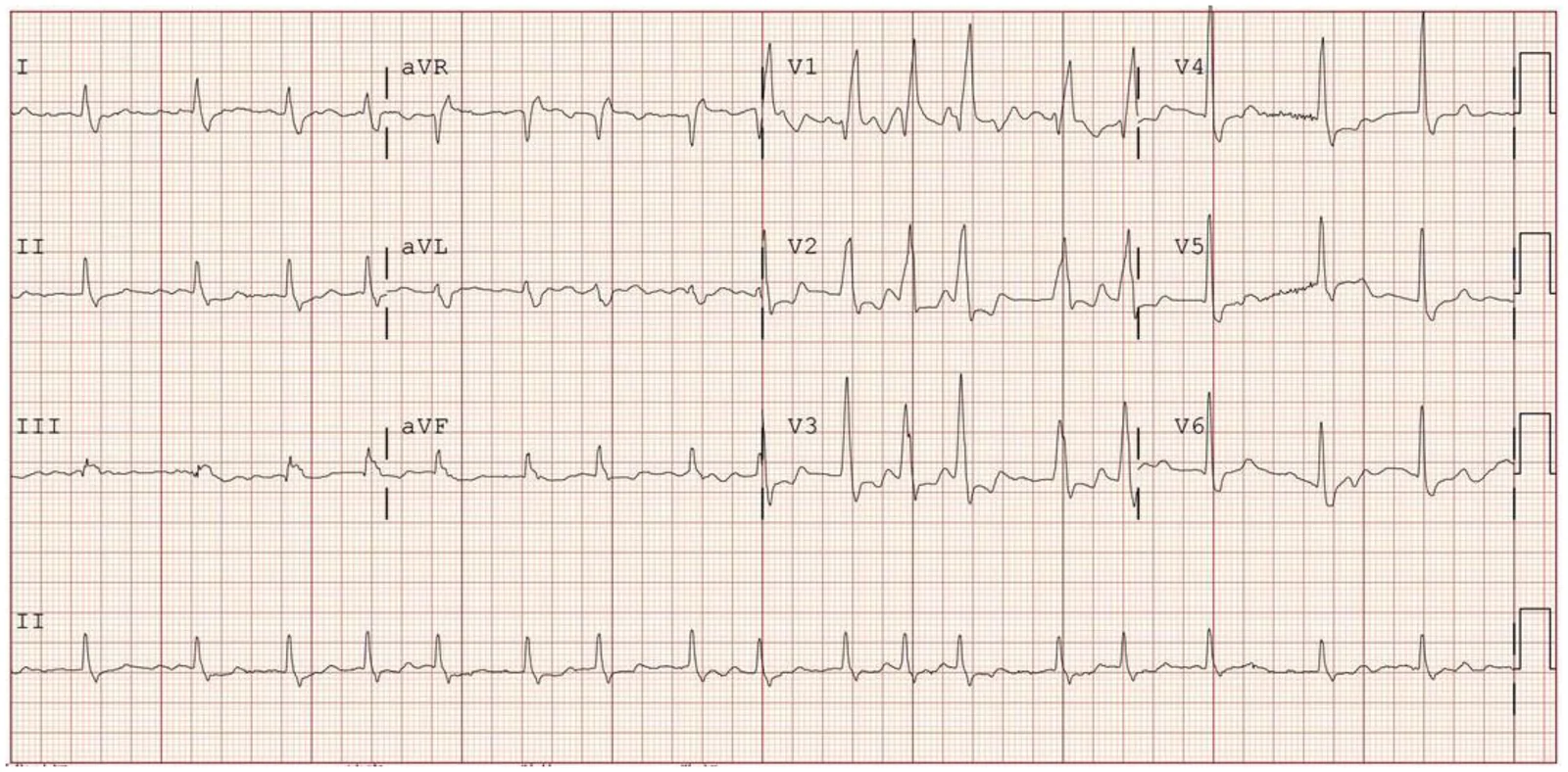
Electrocardiogram on admission. This revealed atrial fibrillation, a complete right bundle branch block, and ST-T segment changes.

Electrocardiography revealed atrial fibrillation, a complete right bundle branch block, and ST-T segment changes (**Figure 1**).

**Figure 2:**
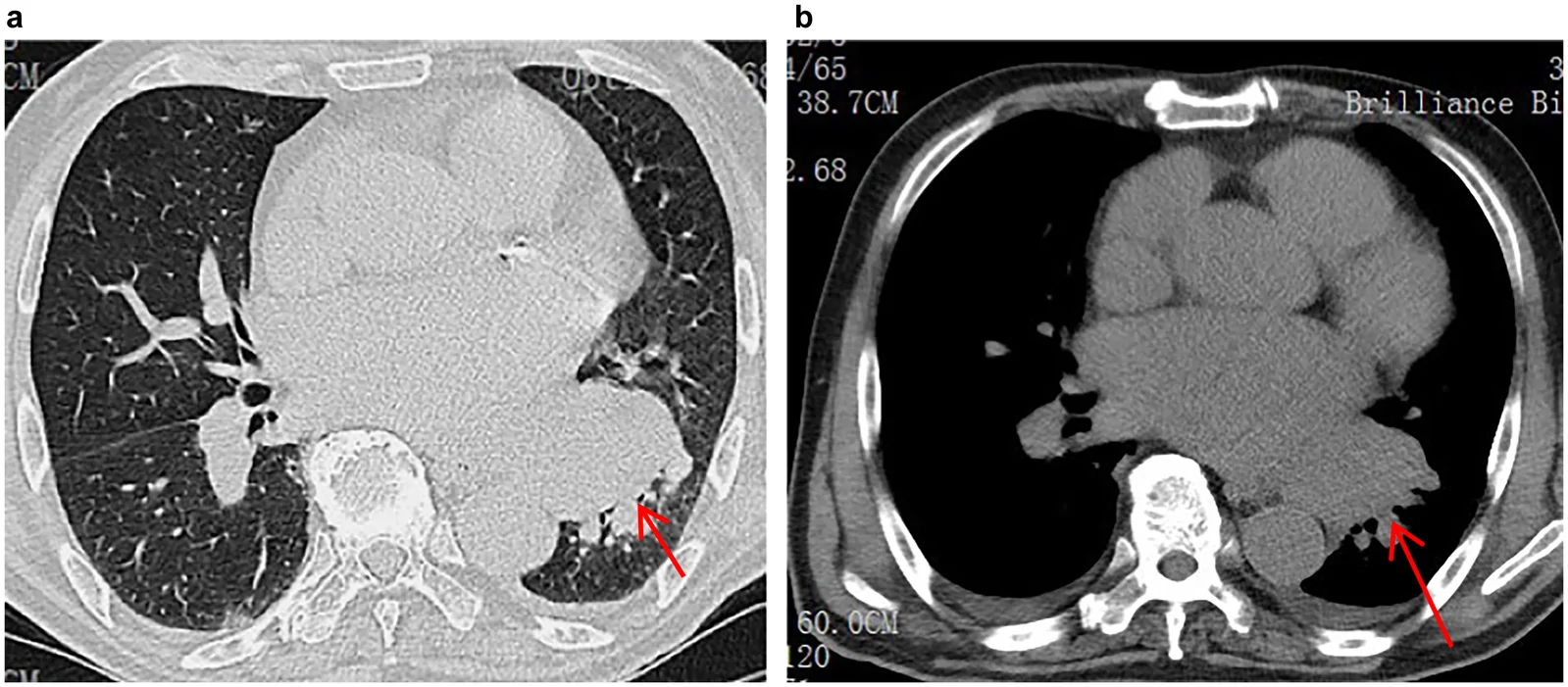
Plain chest computed tomography (CT) images. **(a)** Chest CT scan lung window. **(b)** Mediastinal window. A soft tissue mass was seen near the hilum of the lower lobe of the left lung. This mass showed unclear borders (red arrow), measured approximately 70 × 45 mm, and demonstrated a CT value of approximately 19–38 HU.

**Figure 3:**
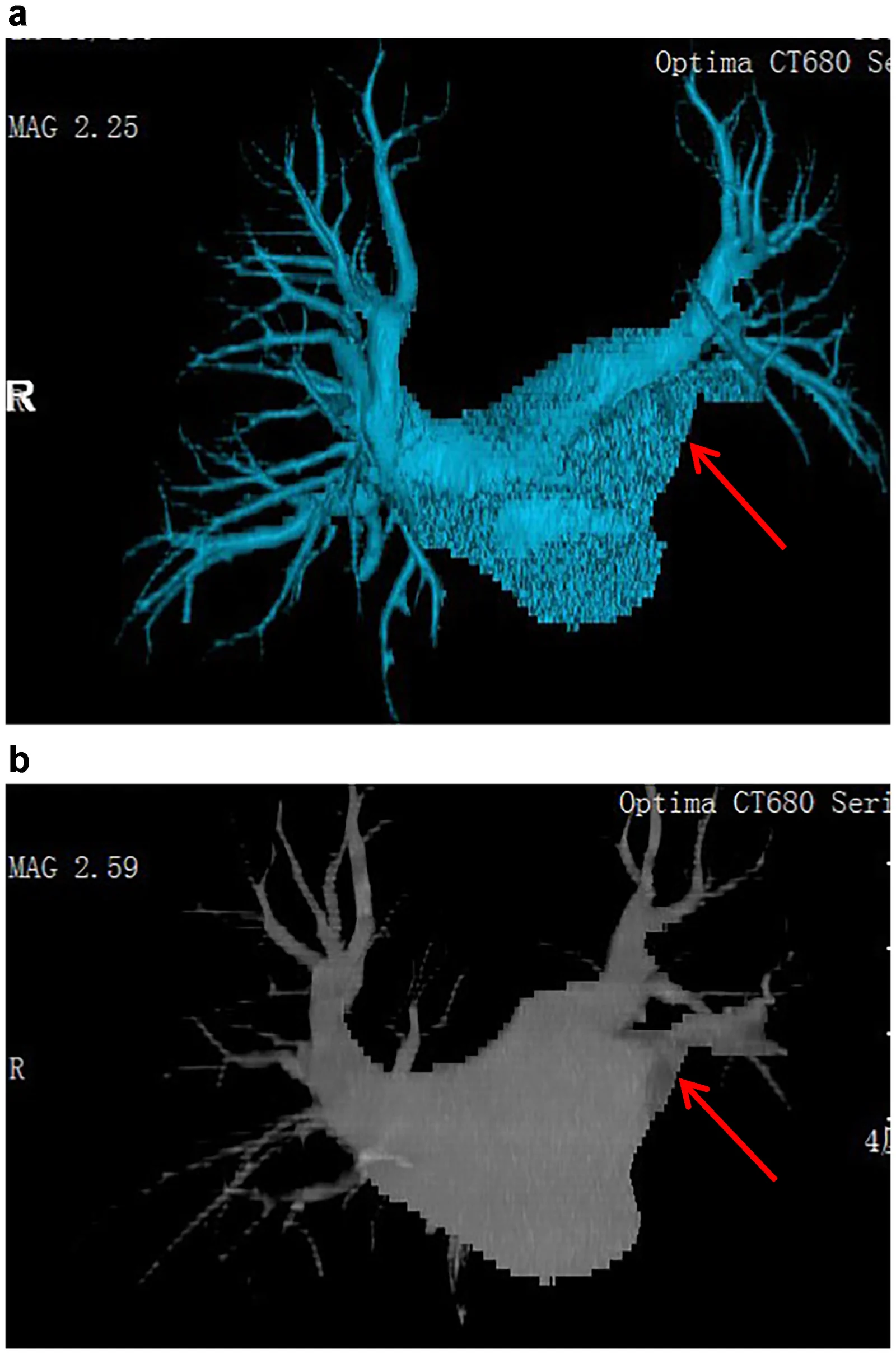
Computed tomography angiography. **(a)** Volume rendering image of the pulmonary veins. **(b)** Maximum intensity projection image of the pulmonary veins. A filling defect shadow was visible in the left inferior pulmonary vein (red arrow).

**Figure 4:**
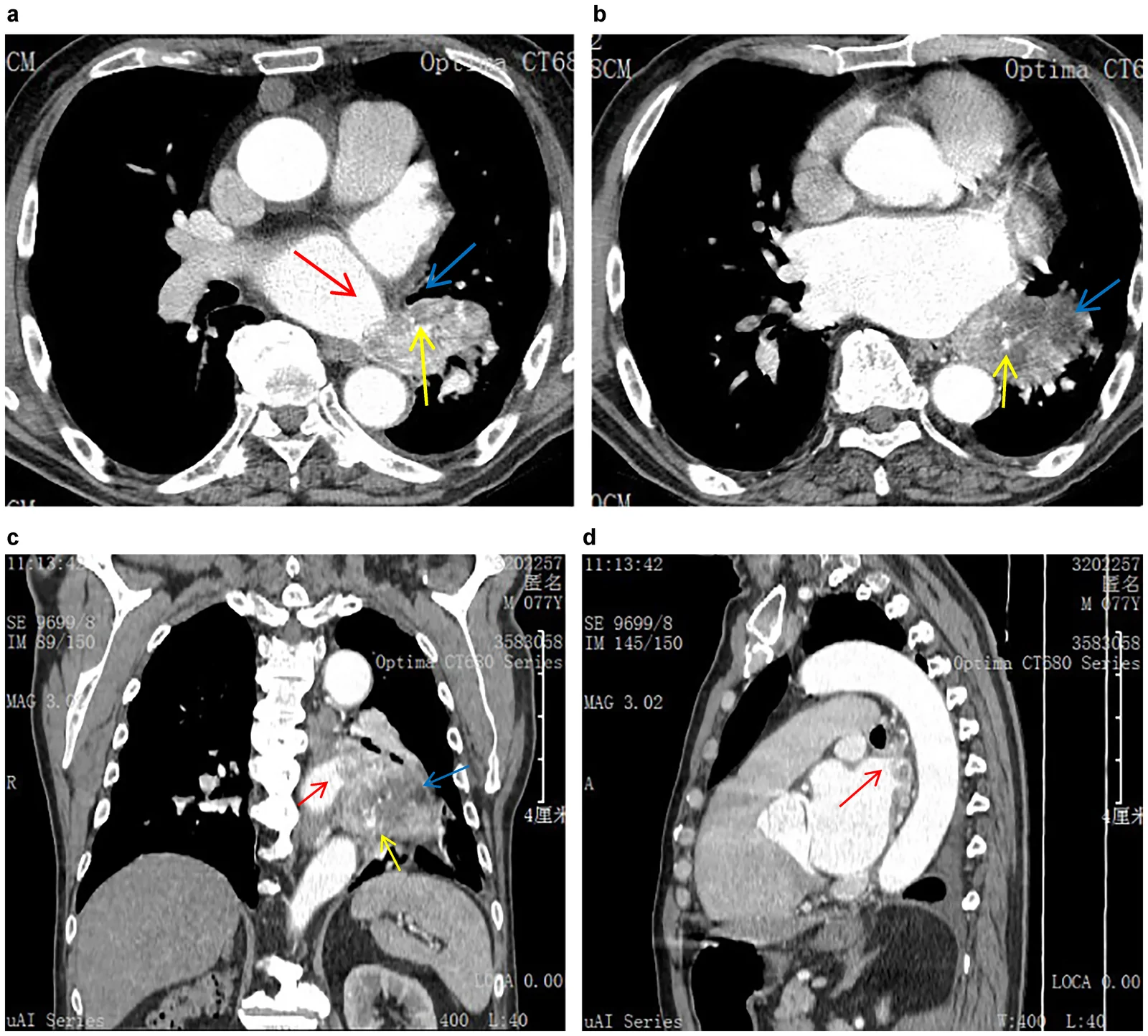
Chest contrast-enhanced computed tomography (CT). **(a, b)** Axially enhanced chest CT scan. **(c)** Coronal. **(d)** Sagittal. Mass-like soft tissue shadow (approximately 70 × 45 mm) was present adjacent to the mediastinum in the lower lobe of the left lung, with unclear boundaries and low-density areas (blue arrow). The average CT value of the lesion was approximately 38 HU, while the low-density region measured approximately 19 HU. The lesion demonstrated heterogeneous enhancement with CT values of approximately 130 HU in the region of marked enhancement and 23 HU in the low-density area. The lesion showed tortuous traversing vascular (yellow arrow) shadowed invading the left pulmonary vein (red arrow).

Plain chest CT showed enlargement of the left pulmonary hilum and possible dilation of the left lower pulmonary artery, and contrast-enhanced CT was recommended (**Figures 2a, b**). CT angiography showed no obvious abnormalities in the pulmonary artery, but a filling defect shadow was observed in the left lower pulmonary vein (**Figures 3a, b**). Chest contrast-enhanced CT revealed a mass-like soft tissue density shadow measuring approximately 70 × 45 mm adjacent to the mediastinum in the left lower lobe, with unclear borders and patchy low-density areas. The average CT value of the lesion was approximately 38 HU, while the low-density region measured approximately 19 HU. The lesion demonstrated heterogeneous enhancement with CT values of approximately 130 HU in the region of marked enhancement and 23 HU in the low-density area. The lesion showed tortuous traversing vascular shadows, adjacent bronchial stenosis and occlusion, invasion of the left pulmonary vein, and multiple mediastinal lymph node enlargements (**Figures 4a–d**). The patient was initially misdiagnosed as having lung cancer.

**Figure 5:**
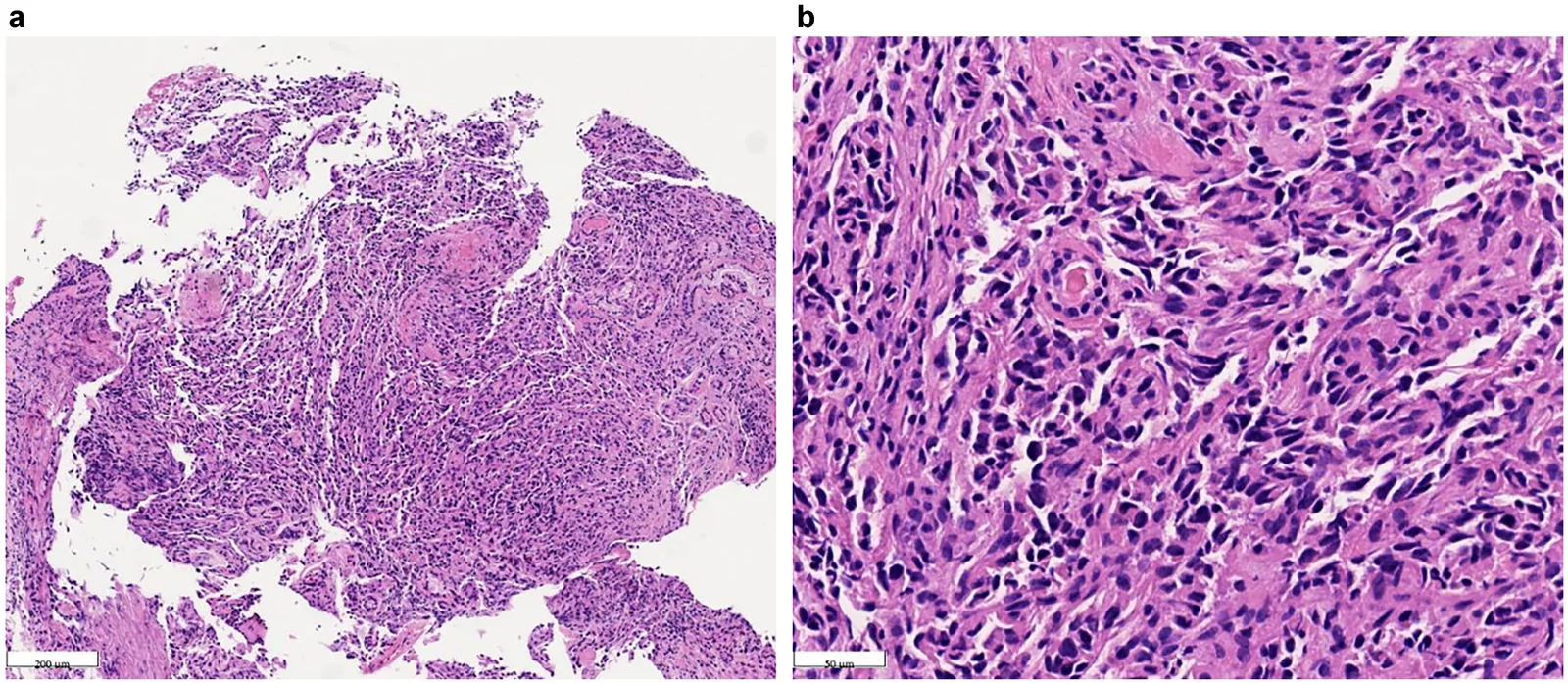
Tumor histology. **(a)** Hematoxylin and eosin staining (×4): Tumor cells are distributed in nodular and whorled patterns, with some normal lung epithelium and glandular structures visible. A small amount of mucus is present in the stroma and no irregular cystic dilated pseudovascular spaces or lymphocyte band structures are observed. **(b)** Hematoxylin and eosin staining (×20): The tumor cells resemble histiocytes, appearing spindle-shaped or ovoid in a whorled arrangement, with rare mitotic figures. The spindle tumor cells have a dense distribution, with hyperchromatic nuclei and an increased nuclear-to-cytoplasmic ratio.

**Figure 6:**
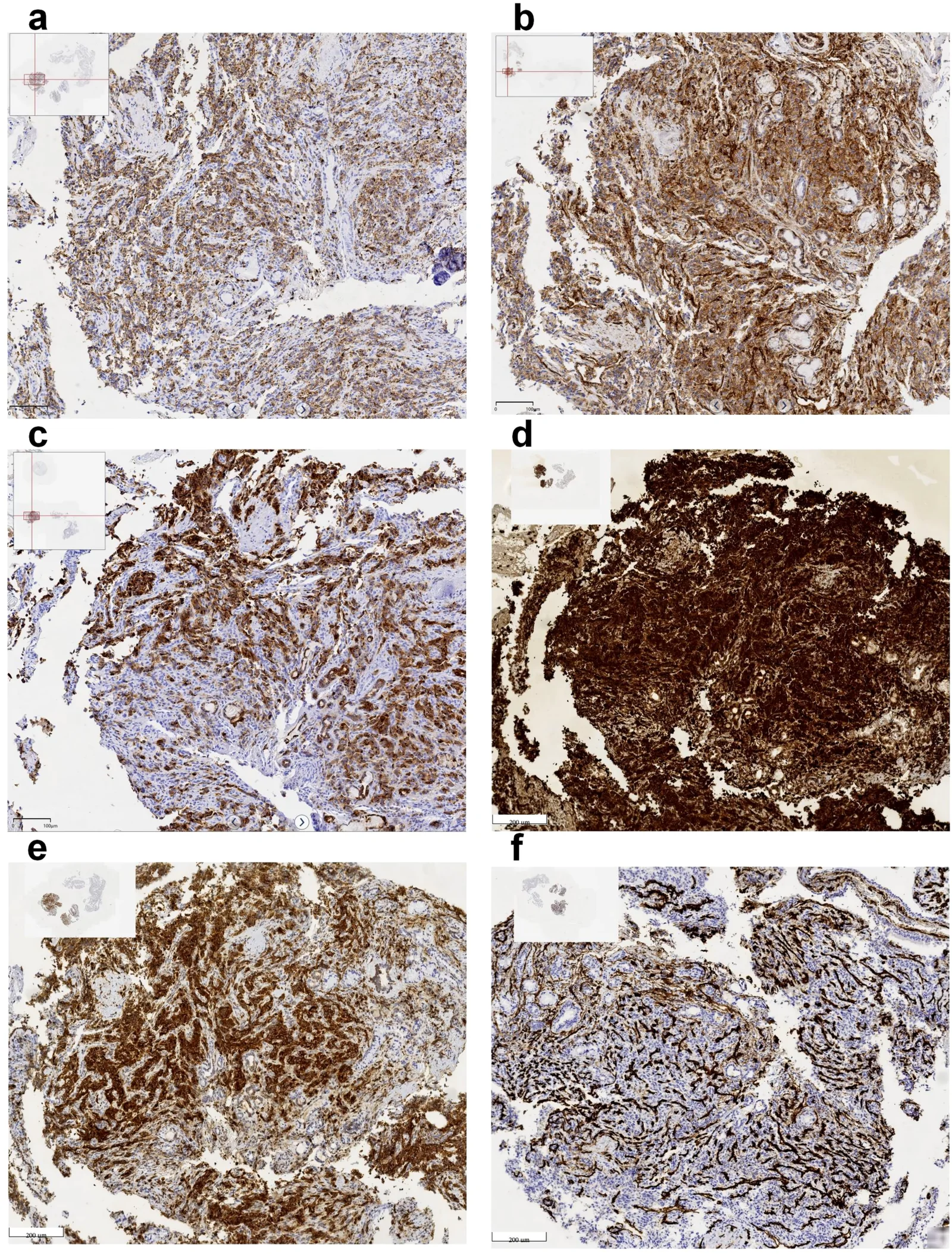
Immunohistochemistry staining of the tumor with positive results. **(a)** CD68(×4). **(b)** CD99(×4). **(c)** EMA(×4). **(d)** Vimentin (×4). **(e)** Bcl-2 (×4). **(f)** CD34 (×4).

**Figure 7:**
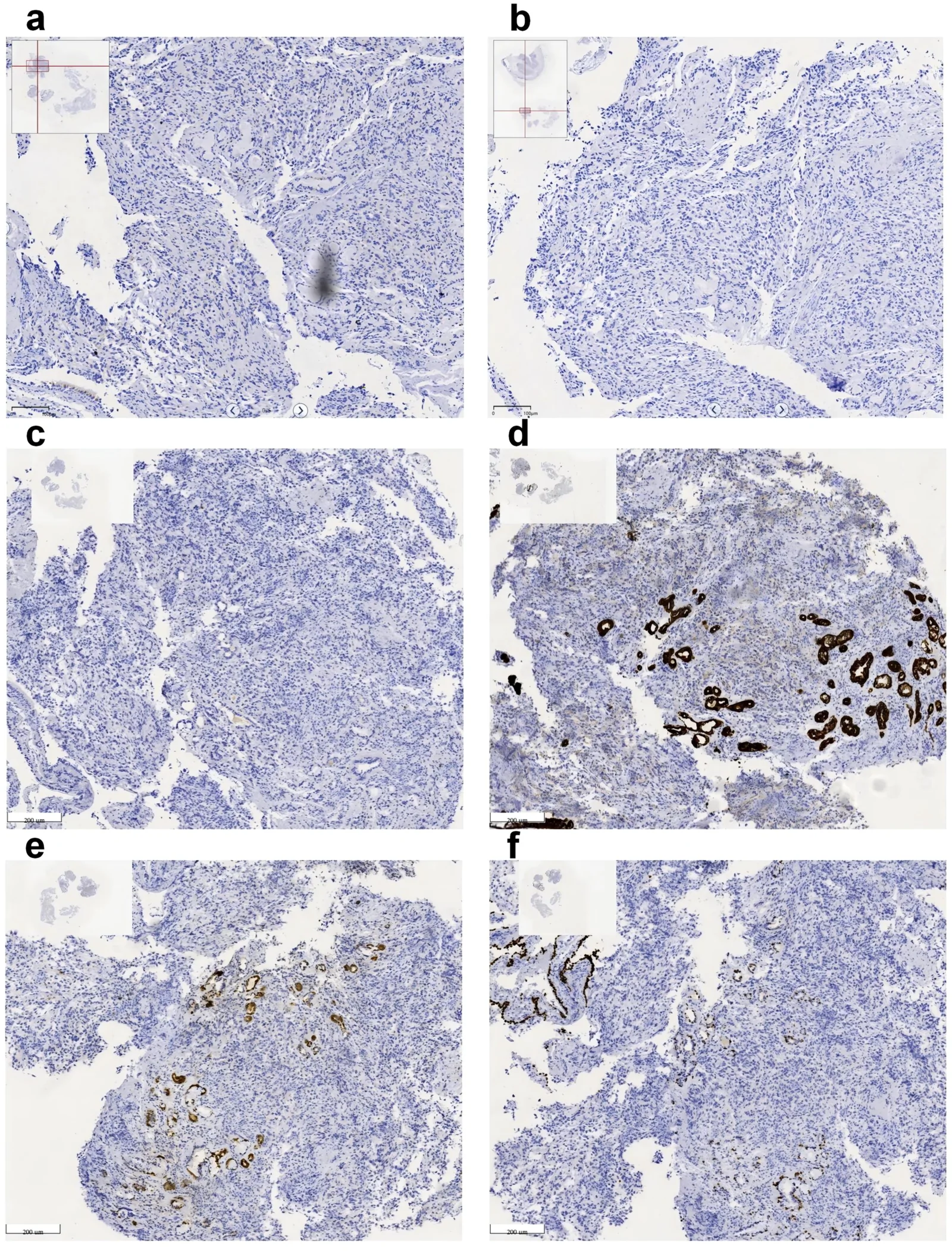
Immunohistochemistry staining of the tumor with negative results. **(a)** STAT6 (×4). **(b)** ALk (×4). **(c)** Calretinin (×4). **(d)** CK (×4). **(e)** S100 (×4). **(f)** P63 (×4).

Bronchoscopy revealed that the lumen of the left lower basal segment was externally compressed, with mucosal swelling. Under low-power magnification, the tumor cells resembled histiocytes or dendritic cells arranged in solid sheets or fascicles with spindle or oval shapes and locally forming whorled patterns. The cytoplasm was pale and eosinophilic, mitotic figures were rare, and myxoid degeneration was observed in the small foci of the tumor stroma. No multifocal hemorrhages or typical pseudoangiomatous lacunar structures were observed (**Figures 5a, b**). This suggested a spindle cell tumor with mild nuclear atypia, consistent with an intermediate-type AFH. Immunohistochemistry results showed Bcl-2 (++), CD56 (+), EMA(+), CD68(+), CD99(+), CD34 (vessels +), SMA (vessels +), Vim (+++), β-Catenin (++), with a Ki-67 index of approximately 10% (**Figures 6a–f**). Other markers such as calretinin, desmin, stat6, alk, TTF-1, CK, S100, and P63 were negative (**Figures 7a–f**).

Due to the limited tissue content of the puncture specimen, genetic testing was not performed in this case. Based on the imaging and pathological findings, the patient was diagnosed with an intermediate-type AFH in the basal segment of the left lower lung lobe. The tumor presented as a mediastinal mass with features of invasion into the adjacent tissues and lymph node enlargement. No abnormalities were observed in the pulmonary artery or other pulmonary vessels, ruling out pulmonary vascular disease.

### 2.3 Therapeutic intervention

Given the patient’s advanced age and multiple underlying diseases, after a full disclosure of the condition and treatment risks, the patient and family declined surgical intervention. Therefore, no surgical treatment was performed for the primary lung tumor, and a supportive and symptomatic management strategy was adopted with plans for close follow-up observation.

### 2.4 Follow-up and outcomes

**Figure 8:**
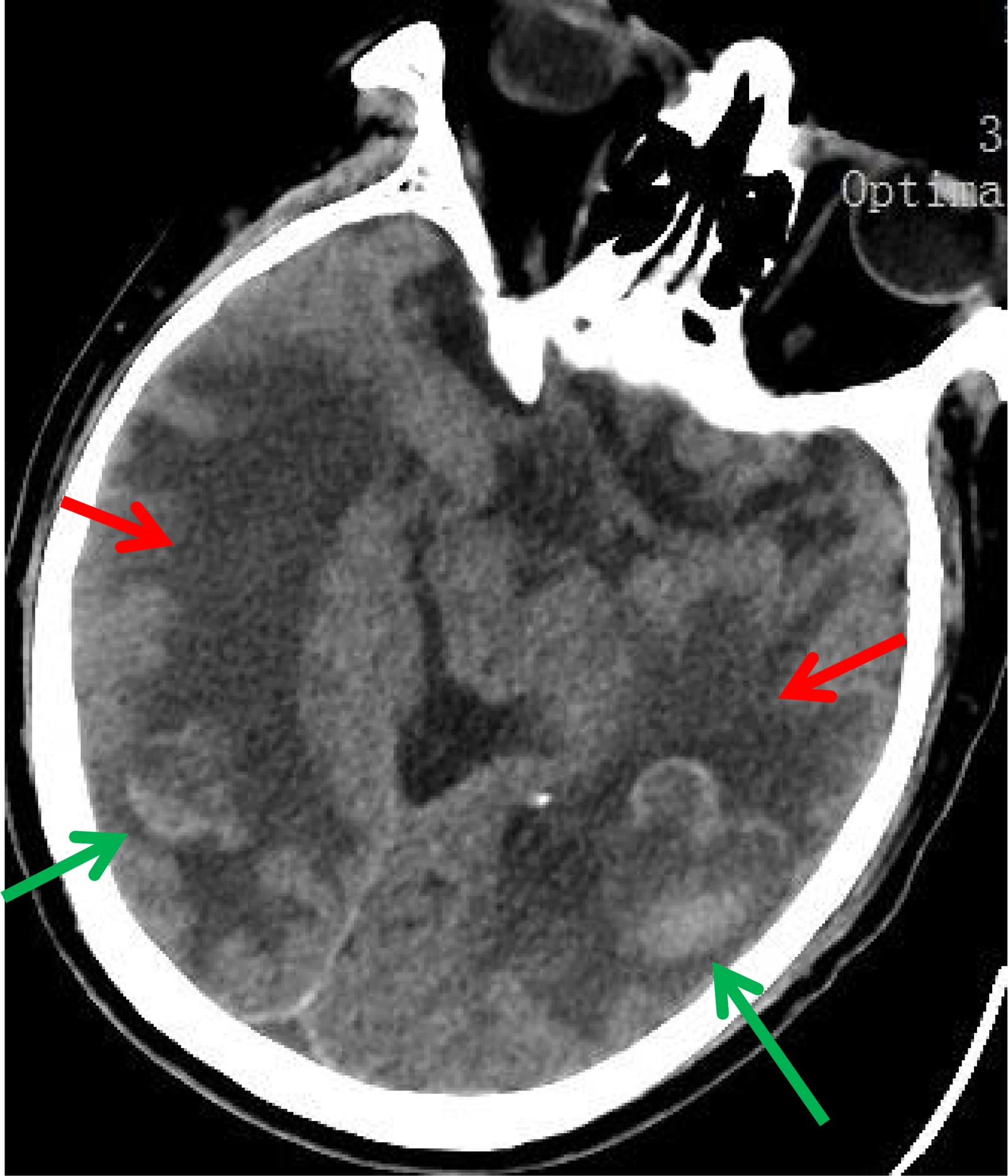
Head computed tomography image. Multiple cystic and solid lesions were present in both cerebral hemispheres, with local hemorrhage (green arrow) and extensive edema (red arrow).

After six months of supportive and symptomatic treatment, the patient developed an intermittent cough, sputum production, worsening chest tightness, shortness of breath, and neurological symptoms, such as dizziness. Head CT revealed multiple cystic-solid lesions in both cerebral hemispheres with local bleeding and extensive edema consistent with brain metastasis, indicating significant disease progression (**Figure 8**). The patient’s condition was complex with a poor prognosis, requiring further comprehensive evaluation and symptomatic management.

## 3 Discussion

We present the case of an older male patient, with the incidental discovery of a left hilar mass following trauma at the initial presentation. These findings were highly suggestive of lung cancer. Subsequent bronchoscopic biopsy and immunohistochemistry confirmed a diagnosis of intermediate-type AFH. Pulmonary AFH is extremely rare. Previously reported cases mostly presented as solitary peripheral pulmonary lesions, with rare instances of obvious hilar vascular and bronchial invasions (9, 10). In this case, imaging showed a mass-like soft tissue shadow with heterogeneous contrast enhancement, internal tortuous vascular shadows, and invasion of adjacent bronchi and pulmonary veins, demonstrating stronger local invasiveness than most AFH cases in the literature. PET/CT imaging holds significant value in the diagnosis of AFH. AFH lesions demonstrate high FDG uptake, and the whole-body imaging capability of PET/CT aids in accurate staging and detection of occult metastases (11). PET/CT was not performed due to family economic constraints in this case, which could have precluded the detection of early metastases, thereby compromising the comprehensiveness of systemic staging and treatment decisions.

The diagnostic process in this case underscores the pitfalls of misdiagnosis in the imaging presentations of pulmonary masses. Clinically, hilar masses are often first considered as common types of lung cancer, especially in older male patients (12). However, the imaging features of pulmonary mesenchymal tumors such as AFH and other rare spindle cell tumors overlap significantly with those of lung cancer. Contrast-enhanced CT may show mass-like soft tissue shadows, heterogeneous enhancement, and vascular structural changes that can lead to misjudgment. Moreover, imaging findings of vascular and bronchial invasion usually suggest highly invasive tumors, but are not specific to lung cancer and can be seen in rare tumors, such as solitary fibrous tumors and hemangiopericytomas (13). Therefore, when dealing with lung masses with imaging findings suggesting high invasiveness but lacking specific tumor markers, the critical role of pathology and immunohistochemistry in the differential diagnosis must be emphasized. AFH exhibits diverse morphological features. The tumor consists of irregularly distributed polygonal, spindle, oval, and round cells with histiocytic or myoid features. The tumor nests exhibit multifocal hemorrhagic cystic spaces devoid of endothelial cell lining, resembling pseudo-vascular cavities or slit-like clefts. A thick and incomplete fibrous pseudo-capsule is often present. The tumor is densely infiltrated by lymphoplasmacytic cells or cystic envelopes, potentially including the formation of germinal center (14). However, the described histologic features are variable; the only consistent finding is the multicellular pattern of oval to spindle cells (15). The histological findings in this case are consistent with previously reported morphological features. AFH generally lacks specific immunohistochemical markers. Some studies have reported that approximately 50% of AFH tumors express desmin, whereas 40–50% of tumors show variable positivity for EMA, CD99, and CD68 (16). In practice, the diagnosis of AFH relies on histological features and specific immunophenotypes. In this case, the AFH tumor cells express EMA, CD68, and CD99, but lack expression of desmin, STAT6, or ALK, which is consistent with the existing literature. The negative expression of these markers is crucial for distinguishing AFH from other spindle cell tumors. The negative expression of STAT6 excluded the diagnosis of solitary fibrous tumor (SFT). As previously reported, a subset of angiomatoid fibrous histiocytoma (AFH) may exhibit aberrant ALK expression, which poses differential diagnostic challenges in distinguishing it from ALK-positive histiocytic proliferations and inflammatory myofibroblastic tumor (IMT) (17). In this case, tumor cells were negative for ALK staining, effectively excluding the diagnosis of IMT. The pathogenesis of this disease remains unclear. When a histological diagnosis is difficult, molecular biological testing is often required. As reported in the literature, molecular detection of Ewing sarcoma breakpoint region 1 (EWSR1) gene rearrangement is positive in more than 90% of cases (18), indicating its significant role in diagnosis. AFH is characterized by the presence of fusion genes resulting from translocation mutations involving three related genes: EWSR1-CREB1, EWSR1-ATF1, and FUS-ATF1. Disruption of the EWSR1 gene is detected in the majority of cases, although other fusion genes may also be present (11). However, the EWSR1 fusion gene is not specific to AFH and occurs in other tumors such as primary pulmonary myxoid sarcoma (PPMS), clear cell sarcoma of soft tissue (CCS) and malignant gastrointestinal neuroectodermal tumor (MGNET) (2, 19). PPMS and AFH both harbor EWSR1 fusion genes and exhibit overlapping morphological features. Immunohistochemistry offers additional diagnostic insight, as AFH often shows positive staining for markers such as CD68 and ALK, which are typically absent in PPMS (20). Owing to the wide range of AFH sites, diverse tissue morphology, and lack of specific immunohistochemical phenotypes, the molecular changes overlap with various tumors, requiring a comprehensive analysis for diagnosis and differential diagnosis (16). The immunohistochemical diagnostic workflow in this case not only avoided the risk of misdiagnosis as common lung cancer but also provided a solid foundation for subsequent treatment strategy formulation.

Additionally, this patient had underlying conditions, including atrial fibrillation, hyperlipidemia, and hyperglycemia, which are uncommon in previous reports on pulmonary AFH. This patient developed multiple brain metastases during the six-month follow-up, with head CT revealing multiple cystic-solid brain metastases and extensive edema. Reports of AFH brain metastasis in the literature are extremely rare. Even in known metastatic-prone mesenchymal tumors like hemangiopericytomas, brain metastasis is uncommon (21, 22). In the context of brain metastasis in angiomatoid fibrous histiocytoma (AFH), gene fusions between EWSR1 and CREB family transcription factors (e.g. ATF1, CREB1) represent a core molecular event. These fusion proteins may induce invasive phenotypes and activate angiogenic pathways through aberrant transcriptional regulation, providing a molecular basis for the metastatic potential of AFH (23). Characterized by an inflammatory phenotype, AFH secretes pro-inflammatory cytokines (e.g. IL-6), which can also disrupt the integrity of the blood-brain barrier by activating signaling pathways (e.g. STAT3) in endothelial cells, thereby promoting the formation of intracranial Metastasis (24). This underscores the importance of not underestimating the risk of distant metastasis from rare tumors, such as pulmonary AFH, particularly among older patients with compromised immune function or complex underlying diseases.

Tumor diagnosis and treatment in older patients are challenging. In cases of localized AFH, complete surgical resection represents the treatment of choice and typically yields favorable local control (25). Underlying conditions, such as atrial fibrillation, hyperlipidemia, and hyperglycemia, increase the risk of surgery and systemic therapy, often limiting treatment options. AFH exhibits a certain sensitivity to radiation therapy. Administration of an adequate radiation dose can effectively control local tumor growth. In a reported case of deep-seated AFH in the popliteal fossa, surgical intervention resulted in only marginal resection due to the large tumor volume and ill-defined boundaries. The patient received postoperative radiotherapy at a total dose of 60 Gy in 30 fractions. One year postoperatively, the patient has remained free of recurrence and metastasis (26). These findings suggest that radical radiation therapy may hold potential for controlling local lesions. In the present case, the patient refused surgery because of advanced age and multiple chronic diseases, which indirectly led to rapid tumor progression and brain metastasis. However, owing to the scarcity of reported cases of AFH with brain metastasis, there is currently no standardized treatment protocol. Clinical decisions often rely on empirical treatment or extrapolation from management strategies for other malignancies. Local treatment modalities, such as surgical resection of brain metastases and radiation therapy, are key to controlling intracranial lesions. Existing literature suggests that for intracranial mesenchymal tumors harboring the EWSR1-ATF1 fusion, surgical resection followed by radiotherapy may help reduce the risk of local recurrence (26). Systemic therapy plays an important role in the management of AFH with brain metastasis, especially when lesions cannot be fully controlled by local modalities or when multiple metastases have occurred. Previous studies have reported that intracranial AFH can induce a systemic inflammatory response mediated by the interleukin-6 (IL-6)/STAT3 signaling pathway, suggesting that interventions targeting this pathway represent a promising therapeutic strategy for managing tumor-related symptoms (24). Unfortunately, despite the availability of multiple therapeutic interventions, our patient was unable to undergo further diagnostic evaluation and treatment secondary to challenges in managing concurrent comorbidities. The diagnosis and treatment process in this case revealed the need for a high degree of vigilance and the establishment of systematic diagnostic and treatment workflows for incidental pulmonary lesions, particularly among older patients with compromised immune function or complex underlying diseases.

This case presents several limitations. Although an abnormal hilar image was found at the initial diagnosis, absence of specific symptoms and the history of trauma delayed timely in-depth follow-up and contrast-enhanced imaging. This postponed tumor diagnosis and resulted in a missed opportunity for early intervention. The patient refused surgery because of advanced age and multiple underlying diseases, preventing complete molecular typing and histological analysis and further limiting the possibility of precision treatment. This patient’s follow-up was limited to six months, resulting in a limited understanding of the tumor’s long-term biological behavior, treatment response, and outcomes. Such constraints mirror the common limitations of rare tumor literature.

Systematic reporting of rare tumor cases not only helps enrich clinical knowledge but also provides a reference for the diagnosis and treatment of similar patients in the future. Future efforts should focus on strengthening multi-center case data accumulation, promoting collaboration among pathology, molecular testing, and clinical multidisciplinary teams, and establishing standardized diagnostic, treatment, and follow-up pathways for rare pulmonary mesenchymal tumors. For older patients or those with complex underlying diseases, a comprehensive management model oriented toward palliation and quality of life should also be explored, aiming to optimize prognosis and enhance the scientific and humanistic aspects of overall medical decision-making.

## Data Availability

The original contributions presented in the study are included in the article/supplementary material. Further inquiries can be directed to the corresponding author.
